# Visualizing multimerization of plasticity-related gene 5 at the plasma membrane using FLIM-FRET

**DOI:** 10.3389/fmolb.2024.1478291

**Published:** 2024-09-30

**Authors:** Franziska Köper, Danara Vonk, Malte W. Dirksen, Isabel Gross, Axel Heep, Torsten Plösch, Mark S. Hipp, Anja U. Bräuer

**Affiliations:** ^1^ Department of Human Medicine, Division of Anatomy, School of Medicine and Health Sciences, Carl von Ossietzky University Oldenburg, Oldenburg, Germany; ^2^ Department of Human Medicine, Division of Perinatal Neurobiology, School of Medicine and Health Science, Carl von Ossietzky University Oldenburg, Oldenburg, Germany; ^3^ Department of Biomedical Sciences, University Groningen, University Medical Centre Groningen, Groningen, Netherlands; ^4^ PicoQuant GmbH, Berlin, Germany; ^5^ University Hospital for Paediatrics and Adolescent Medicine, Oldenburg, Germany; ^6^ Research Center of Neurosensory Science, Carl von Ossietzky University Oldenburg, Oldenburg, Germany; ^7^ Department of Obstetrics and Gynaecology, University Medical Center Groningen, University of Groningen, Groningen, Netherlands; ^8^ School of Medicine and Health Sciences, Carl von Ossietzky University Oldenburg, Oldenburg, Germany

**Keywords:** FLIM-FRET, plasticity-related genes, protein-protein interaction, live-cell imaging, protrusions, neuronal plasticity, oligomerization, primary neurons

## Abstract

Plasticity-related gene (PRG) 5 is a vertebrate specific membrane protein, that belongs to the family of lipid-phosphate phosphatases (LPPs). It is prominently expressed in neurons and is involved in cellular processes such as growth-cone guidance and spine formation. At a functional level, PRG5 induces filopodia in non-neuronal cell lines, as well as the formation of plasma membrane protrusions in primary cortical and hippocampal neurons. Overexpression of PRG5 in immature neurons leads to the induction of spine-like structures, and regulates spine density and morphology in mature neurons. Understanding spine formation is pivotal, as spine abnormalities are associated with numerous neurological disorders. Although the importance of PRG5 in neuronal function is evident, the precise mechanisms as to how exactly it induces membrane protrusions and orchestrates cellular processes remain unresolved. Here we used *in vitro* biochemical assays to demonstrate that in HEK293T cells a large fraction of PRG5 can be found in homo dimers and lager multimers. By using Fluorescence Lifetime Imaging (FLIM) to quantify Förster Resonance Energy Transfer (FRET), we were able to visualize and quantify the specific localization of PRG5 multimers in living HEK293T cells and in fixed immature primary hippocampal neurons. Here, we provide the first evidence that PRG5 multimers are specifically localized in non-neuronal filopodia, as well as in neuronal spine-like structures. Our findings indicate a potential functional role for PRG5 multimerization, which might be required for interaction with extracellular matrix molecules or for maintaining the stability of membrane protrusions.

## 1 Introduction

Brain development is a highly orchestrated process that begins with the differentiation of neuronal precursor cells continuing until late adolescence. It involves a series of complex, dynamic, and adaptive processes that promote the formation and differentiation of neuronal structures (Stiles and Jernigan, 2010). In the central nervous system, dendritic spines are small, actin-rich protrusions that emerge from the dendritic shafts of neurons (Yoshihara et al., 2009). These structures are initially formed during the early postnatal period and persist until adulthood. Furthermore, they exhibit a wide range of sizes and shapes, allowing for a high degree of plasticity (Hering and Sheng, 2001; Tashiro and Yuste, 2003; Yuste and Bonhoeffer, 2004). While the pivotal role of dendritic spines in synaptic transmission and plasticity is well established, the precise mechanisms underlying spine formation and functionality are not fully understood (Ethell and Pasquale, 2005; Yoshihara et al., 2009; Runge et al., 2020). So far, several molecules and proteins involved in neuronal spinogenesis have been identified (Zhang and Benson, 2000; Arimura and Kaibuchi, 2005; Sala et al., 2008). One of them is plasticity-related gene (PRG) 5, which is expressed in a vertebrate- and neuron-specific manner (Coiro et al., 2014).

To date, five PRGs proteins (PRG1-5) have been identified. They all consist of six transmembrane domains, three extracellular loops, and intracellular N- and C-termini (Strauss and Bräuer, 2013; Brandt et al., 2024). PRGs show homology to lipid-phosphate phosphatases (LPP), which are enzymes that dephosphorylate bioactive lipids such as lysophosphatidic acid (LPA). However, PRGs have non-conservative substitutions in their catalytic domain, which is why they lack enzymatic activity and therefore comprise a new subfamily (Sigal et al., 2005; Bräuer and Nitsch, 2008; Brindley and Pilquil, 2009; Strauss and Bräuer, 2013). PRG5 (synonyms: LPPR5, PLPPR5, PAP2D) mRNA and protein is dynamically expressed throughout the brain in areas of high plasticity (e.g., hippocampus, cerebellum, bulbs), as well as in peripheral tissues (e.g., kidney, testis, placenta) (see Fuchs et al., 2022; Brandt et al., 2024 for a comprehensive review). At a functional level, PRG5 is involved in the formation of filopodia in various cell types, including N1E-115 neuroblastoma, P19 carcinoma and HEK293T cells, as well as in the formation of plasma membrane protrusions in primary cortical and hippocampal neurons (Broggini et al., 2010; Gross et al., 2022b). In immature neurons, PRG5 overexpression leads to an early formation of spine-like membrane structures, while it regulates spine density and morphology in mature neurons. Downregulation of PRG5 results in a loss of excitatory synapses and consequently in reduced neuronal functionality (Coiro et al., 2014). Furthermore, PRG5 knock-out mice show reduced seizure latency, increased seizure susceptibility, as well as aberrant mossy fiber sprouting (Wang et al., 2021). These results indicate that PRG5 plays an important role in dendritic spine development and has a potential neuroprotective role (Gross et al., 2022a; Wang et al., 2022). Nevertheless, exactly how PRG5 is involved in the formation and/or stabilization of dendritic spines remains the subject of ongoing investigations. One possible mechanism that may underly the functionality of PRG5, is protein-protein interaction (PPI). PPIs play a crucial role in regulating a multitude of molecular and cellular functions, including signal transduction and the organization of cellular structures (Jones and Thornton, 1996; Braun and Gingras, 2012). Understanding PPIs in their cellular context is a crucial step towards a better understanding of the complex biological functions of proteins. Of note, PPIs have long been known in LPPs, which belong to the family of phosphatases and phosphotransferases, and consist of three related members (LPP 1–3) (Sigal et al., 2005; Tang et al., 2015). In contrast to PRGs, they possess functional catalytic domains in their structure, which allows them to dephosphorylate bioactive lipids in the extracellular space. Notably, LPPs can form both homo and hetero dimers (Burnett et al., 2004; Long et al., 2008). Multimerization seems to occur commonly among LPP members. For example, the *Drosophila* LPP homologue Wunen forms only homo dimers, but not hetero dimers, with Wunen2 or human LPP1 and LLP3. Although it was shown that C-terminal truncation or mutations within the catalytic domain prevented the association, dimerization was not required for activity *in vitro* or *in vivo* (Burnett et al., 2004). However, it is hypothesized that these different combinations of oligomeric states might regulate subcellular localization (Long et al., 2008). The first evidence that PRG5 forms complexes with other PRG members was reported by Yu and colleagues in 2015. Using co-immunoprecipitation and co-localization studies, they show cooperative interactions of PRG5 with its family members PRG2 and PRG3 (Yu et al., 2015). PRG5 homo dimerization was first shown by Gross and colleagues in 2022. Western blot analysis of isolated PRG5 from overexpressing HEK293H cells revealed that PRG5 is present not only as monomeric but also as higher molecular weight bands, indicating possible multimeric forms. This finding was subsequently validated using mass spectrometry, which confirmed the presence of PRG5 in these higher molecular weight bands. Furthermore, analysis of endogenous PRG5 protein lysates from the cerebellum, cortex, hippocampus, and bulbus demonstrated the presence of similar higher multimeric bands (Gross et al., 2022a).

We hypothesize that PRG5 forms functional multimers at the plasma membrane. By using *in vitro* solubility and deglycosylation assays, we demonstrated that both monomeric and multimeric version of PRG5 were present in a soluble form and are N-glycosylated. We proved that PRG5-enhanced green fluorescent protein (eGFP), but not eGFP alone, was able to co-precipitate with PRG5-FLAG, confirming the existence of at least PRG5 homo dimers. Further investigations involved the use of Fluorescence Lifetime Imaging (FLIM) to quantify the extent of Förster Resonance Energy Transfer (FRET) between two PRG5 proteins labeled with fluorescent molecules that served as a proxy for direct protein-protein interaction. FRET is a physical, nonradiative process that occurs when an excited fluorophore (donor) and a second fluorophore (acceptor) come into close proximity (<10 nm) (Vogel et al., 2006; Periasamy et al., 2015; Algar et al., 2019). FLIM is a technique that enables the observation and quantification of changes in the amount of FRET (Liput et al., 2020). FLIM-FRET is not affected by variations in fluorophore concentration or excitation intensities, and allows the visualization of interactions with high tempo-spatial resolution, which makes it the method of choice for reliable detection of PPIs (Chen et al., 2003; Wallrabe and Periasamy, 2005; Fritz et al., 2010; El Meshri et al., 2015). By using FLIM-FRET we were able to show that PRG5 multimerization takes place in plasma membrane filopodia in living HEK293T cells. Notably, these results were further confirmed in immature primary hippocampal neurons, where PRG5 multimers are enriched at the tip of spine-like membrane structures. These findings indicate a potential functional role for PRG5 multimerization, which might be required for interaction with other molecules or for maintaining the stability of plasma membrane protrusions.

## 2 Materials and methods

### 2.1 Animals

Timed-pregnant mice (C57BL/6J) were obtained from the central animal facility of the Carl von Ossietzky University Oldenburg Germany. The animals were kept under standard laboratory conditions in accordance with German and European guidelines for the use of laboratory animals (12 h light/dark cycle; 55% ± 15% humidity, 24°C ± 2°C room temperature (RT) and water and food *ad libitum*). All experiments were performed in accordance with the institutional guidelines for animal welfare and approved by the “Niedersächsisches Landesamt für Verbraucherschutz und Lebensmittelsicherheit” (33.19-42502-04-22-00234). Embryonic day 18 mice were used for culturing primary hippocampal neurons. For determining embryonic stages, the day of the vaginal plug following mating was assigned as embryonic day 0.5.

### 2.2 Plasmids

To examine the possible influence of fluorophore localization on the FLIM-FRET results, donor (monomeric Turquoise, mTurquoise) and acceptor (enhanced yellow fluorescent protein, eYFP) were fused to either the C-terminus or the N-terminus of PRG5. For the C-terminal constructs, *Prg5* was amplified by PCR using the primers listed in Table 1 and PRG5-eGFP as the template (Broggini et al., 2010; Coiro et al., 2014). PCR products were purified by agarose gel electrophoresis and the QIAEX II Gel extraction kit (Qiagen, Hilden, Germany). *Prg5* was cloned into the respective vectors (eYFP-N1, mTurquoise-N1) via digestion with the respective enzymes: HindIII and BamHI for PRG5-eYFP and PRG5-mTurquoise, (all enzymes from Thermo Fisher Scientific, Walthman, MA, United States). A different strategy was used for the N-terminal constructs. It is known that the fusion of eGFP to the N-terminus of PRG5 prevents its localization to the membrane and, consequently, the induction of filopodia (Gross et al., 2022b). Given that murine PRG5 (Uniport identification number: Q8BJ52) has an extremely short N-terminus, comprising only five amino acids (Gross et al., 2022b), it is possible that a tag positioned too closely to effectively interact with PRG5. This could potentially disrupt the correct protein folding and/or insertion into the membrane (Chen et al., 2013). To address this issue, a new construct was designed that incorporated a spacer sequence (ggttcagctggcagcgctgcaggtagcggagagttc) between eGFP and the N-terminus of PRG5. This spacer sequence was designed by Waldo and colleagues express GFP- fusion proteins for rapid protein folding assay (Waldo et al., 1999). The incorporation of spacer sequences is a standard technique employed to enhance the expression of fusion proteins. Such incorporation maintains the required distance between the proteins, thus allowing for independent folding. Moreover, spacer sequences can enhance the stability and bioactivity of fusion proteins (Chen et al., 2013). In contrast to the original N-terminal construct (Gross et al., 2022b), the insertion of a spacer sequence enabled the plasma membrane transport of PRG5 and thus subsequent filopodia induction. To do so, we first amplified *Prg5* by PCR using the primers listed in Table 1. The PCR product was purified and cloned into the empty GFP-C1 vector using BamHI and EcoRI (Thermo Fisher Scientific). Next, the oligos used to generate the spacer sequence were annealed using the primers in Table 1, phosphorylated using the Anza™ T4 Polynucleotide kinase Kit (Thermo Fisher Scientific), and subsequently purified and inserted using Bglll and HindIII (Thermo Fisher Scientific). For FLIM-FRET, eGFP-C1 was exchanged against eYFP-C1 and mTurquoise-C1 using AgeI and BglII (Thermo Fisher Scientific). The resulting plasmids were verified by sequencing (Eurofins genomics, Ebersberg, Germany).

**TABLE 1:** Construct overview. Plasmids previously generated are listed with their source, while plasmids designed in this study include information of used the primers in 5’ - 3′ direction.

Plasmid	Source	Forward primer	Reverse primer
eGFP-C1	Clontech/Takara Holdings Shimogyo-ku, Kyoto, Japan		
CFP-MEM	Clontech/Takara Holdings		
eYFP-C1	Clontech/Takara Holings		
eYFP-N1	BD bioscience, Franklin lakes, NY, United States		
PRG5-eGFP	Broggini et al. (2010), Coiro et al. (2014)		
PRG5-FLAG	Coiro et al. (2014)		
mTurquoise-C1	Addgene plasmid 60558 (Watertown, MA, United States) (Goedhart et al., 2010)		
mTurquoise-N1	Addgene plasmid 60559 (Goedhart et al., 2010)		
PRG5-peYFP		ttattcaagcttatgcccctgctg	ttattcggatccattgtgacttctgc
PRG5-pmTurquoise		ttattcgaattcatgcccctgctg	ttattcggatccattgtgacttctgc
eGFP-PRG5 PRG5 primer		tactgagaattcacccctgctgccc	gaccgaggatcctgtgacttccgcaaa
eGFP-PRG5 Spacer primer		gatctggttcagctggcagcgctgcaggtagcggagagttcgacaa	agctttgtcgaactctccgctacctgcagcgctgccagctgaacca

### 2.3 HEK293T cell culture and transfection

HEK293T cells were maintained in Dublecco’s Modifed Egal Medium (DEMEM; Thermo Fisher Scientific) supplemented with 10% fetal bovine serum (FBS, PAN-Biotech, Aidenbach, Germany), 2 mM L-gluthamine (Merck, Darmstadt, Germany), 100 units/mL penicillin, and 100 μg/mL streptomycin (PAN-Biotech) under standard conditions of 5% (v/v) CO_2_ and 37°C. Cells were passaged as they reached 90% confluency. Cells were free of mycoplasm, as confirmed by regular testing. For FLIM-FRET analysis, cells were seeded in 35 mm poly-d-lysine coated Petri dishes (FluoroDish, World precision instruments, Sarasota, United States) with 0.2 × 10^6^ cells and transfected after 24 h with polyethyleneimine (PEI, Polysciences, Warrington, PA, United States). Constructs used for transfection are listed in Table 1. For FLIM-FRET measurements, 0.5 µg DNA per dish was used if one construct was transfected, while for co-transfection of two constructs 0.25 µg DNA of each plasmid was used. The DNA was mixed with 50 µL of serum free DMEM. In a second tube, 0.5 µg PEI was added to 50 µL serum free medium. Both tubes were mixed, incubated for 30 min at RT and added to the cells. For Western blot, 2 × 10^6^ cells were seeded in 100 mm Petri dishes and transfected 24 h after using PEI (5 µg DNA for single transfection, 2.5 µg for double transfection, 10 µg PEI and 800 µL serum-free medium per dish). Analyses were continued 14–18 h after transfection.

### 2.4 *In vitro* biochemical assays: solubility assay, deglycosylation assay and Co-immunoprecipitation

For the solubility assay, cells were harvested in a buffer containing 50 mM Tris (pH 7.4), 150 mM NaCl, 1% Triton X-100, 0.1% SDS, and 5 mM EDTA. Cells were lysed by sonication for 3 times 3 s at 50% amplitude. Lysates were incubated on ice for 30 min and subsequently centrifuged for 10 min at 10.000 x g at 4°C and the supernatant collected. 1,650 μg of protein was added to a total volume of 200 μL, and a sample to use as whole cell lysate was collected from this mixture. The remaining sample was centrifuged for 45 min at maximum speed at RT. The supernatant was collected and the pellet resuspended in 1x Laemli buffer (0.5% SDS, 10% Glycerol, 60 mM Tris (pH 6.8), 0.01% bromophenol blue, 5% β-mercaptoethanol). For the deglycosylation assay, cells were harvested in a buffer containing 50 mM Tris (pH 7.4), 150 mM NaCl, 1% Triton X-100, 0.1% SDS and 5 mM EDTA. Cells were lysed as described and the supernatant collected. 25 μg of protein was subjected to deglycosylation using the Peptide-N-Glycosidase F (PNGase F) Glycan Cleavage kit (Gibco, USA) according to the manufacturer’s instructions. For co-immunoprecipitation, HEK293T cells were lysed 19 h after transfection in a detergent buffer [150 mM NaCl, 1% Ecosurf™ EH-9, 50 mM Tris HCl (pH 8.0)] containing protease inhibitors (Merck). Subsequently, eGFP or FLAG fusion proteins (Table 1) were purified using either µMACS GFP or FLAG isolation kits (Miltenyi Biotec, Bergisch Gladbach, Germany) according to the manufacturer’s instructions. For the solubility and the deglycosylation assay, samples were incubated at 55°C for 5 min after the addition of Laemli buffer, while for the co-immunoprecipitation samples were incubated at 95°C for 5 min. For protein analysis, samples were loaded on an SDS-PAGE gel and further transferred on a nitrocellulose membrane using the tank-blotting procedure. Non-specific antibody binding was blocked with 5% (w/v) milk (Carl Roth, Karlsruhe, Germany) in Tris-buffered saline with Tween 20 (TBST). Primary antibodies were incubated overnight at 4°C in blocking solution [mouse-anti-GFP (JL-8) 1:2.500 (abcam, Cambridge, United Kingdom), mouse-anti-GAPDH (1:5.000) (Fitzgerald, United States); rabbit-anti-FLAG 1:2.500 (Abcam); rabbit-anti-PPIA 1:1.500 (Abcam)]. Afterwards, membranes were washed three times with TBST, and subsequently incubated with secondary antibodies in blocking solution for 1.5 h at RT (Goat-anti-Mouse-Alexa 488 (Thermo Fisher Scientific) 1:2.000, Amersham ECL Rabbit IgG, horseradish-peroxidase linked antibody (GE Healthcare, Chicago, IL, United States) 1:10.000). Membranes were again washed three times and any immunoreaction was detected with Clarity ECL substrate (Bio-Rad Laboratories, Hercules, CA, United States) according to the manufacturer’s protocol.

### 2.5 Primary mouse hippocampal neuron culture

Hippocampi of all embryonic day 18 (±0.5 days) mouse embryos from one pregnant mouse were collected, pooled and washed as previously described (Brewer et al., 1993). Neurons were plated onto poly-l-lysine coated glass cover slips (Epredia, Kalamazoo, MI, United States) at a density of 0.9 × 10^6^ cells/well in Minimal Essential medium (Thermo Fisher Scientific) supplemented with 0.6% glucose, 10% (v/v) horse serum (Gibco/Thermo Fisher Scientific), and 100 units/mL penicillin/streptomycin (PAN-Biotech). 3 h after plating, the medium was changed to Neurobasal A medium, supplemented with 2% (v/v) B27 (both from Gibco/Thermo Fisher Scientific), 0.5 mM glutamine (Merck) and 100 units/mL penicillin/streptomycin (PAN-Biotec). Neurons were routinely maintained at 37°C and 5% (v/v) CO_2_. At days *in vitro* (DIV) 1.5, neurons were transfected with the respective plasmids eYFP-C1, mTurquoise-C1, PRG5-mTurquoise, and PRG5-eYFP (Table 1) using Effectene (Qiagen) according to the manufacturer’s instructions. At DIV 2, neurons were fixed in 4% (w/v) PFA and 15% sucrose in PBS for 10 min at RT. Coverslips were washed three times with PBS and mounted on microscopy slides using Mowiol/DABCO.

### 2.6 FLIM-FRET measurement

Directly before the live-cell FLIM-FRET measurements, the medium of transfected HEK293T cells was replaced by DMEM without phenolred (PAN-biotech). For HEK293T cells and neurons, an inverted confocal laser scanning FV3000 microscope (Olympus/Evident, Shinjuku, Japan) with a time resolved laser scanning microscope (LSM) upgrade kit (PicoQuant GmbH, Berlin, Germany) was used. All measurements were conducted with a ×40 oil-immersed objective (UplanXApo, Olympus/Evident, numerical aperture 1.4) at 37°C, and in 5% CO_2_. Each cell was located and focused using confocal lasers (Olympus/Evident). In the case of single transfected cells, a 445 nm laser was used to visualize mTurquoise transfected cells, while in the case of double transfected cells, an additional 514 nm laser was used to visualize the success of the mTurquoise/eYFP double transfection. Unlike conventional FRET, FLIM-FRET measures only the changes in fluorescence lifetime of the donor. Therefore, cells were illuminated using a pulsed 443 nm laser diode (LDH-D-C 440, PicoQuant GmbH) at 25 mHz pulse frequency. Fluorescence emission was detected with a PMA Hybrid-40 (PicoQuant GmbH) detector below 482/35 nm by means of a specific bandpass filter (H488 lpxr, AHF Analysetechnik, AG, Tübingen, Germany). Image frame size was 512 × 512 pixel and the time-correlated singe photon counting resolution was 10.0 picoseconds.

### 2.7 FLIM-FRET analysis

Fluorescence lifetime images were analyzed using the SymPhoTime 64bit software (PicoQuant GmbH). In the software, the distribution decay was further analyzed with a non-linear least-square interactive procedure. To achieve an adequate fit of the lifetime decay of the donor, it was assumed that decays result from a sum of two exponential terms. It is known for various fluorescent proteins that in living cells they adopt different conformational states with varying fluorescent properties, and these have to be considered in the equation (Liput et al., 2020). Therefore, a two exponential fit was applied. For every cell, the amplitude-weighted average lifetime was calculated according to the following equation:

$$\tau_{amplitude}=\frac{\sum_{i}a_{i\;\tau i}}{\sum_{i}a_{i}}$$

The parameter $a_{i}$ is the amplitude of the i -th exponential component, while $\tau_{i}$ is the corresponding lifetime of the i -th exponential component. The lifetime fit was judged by χ^2^ and its residuals. This value represents the differences between the measured fluorescence decay and the calculated decay function, and was always <1.2 (Liput et al., 2020).

FRET efficiency was calculated according to the following equation:

$$E\left(\%\right)=1\;\frac{\tau DA}{\tau \text{DO}}\;x\;100$$

Here, the lifetime was either measured from cells only expressing the donor protein (Donor only = DO) or cells that were co-transfected with the donor and acceptor protein (Donor + acceptor = DA).

### 2.8 Statistics/data analysis

Data analysis was performed using GraphPad Prism 7.05 (GraphPad Software Inc., La Jolla, CA, United States). All data is presented as mean ± SEM. A level of confidence of *p* ≤ 0.05 was adopted. If required *p*-values for the individual experiments are listed in the Supplementary Tables S2–S11. Values were analyzed for normal distribution using the Shapiro Wilk and Pearson test. In the case of normally distributed data, analysis was performed using 1-way ANOVA [robust against violation of normal distribution (Schmider et al., 2010)] followed by Tukey’s or Sidak’s multiple comparison test, with a single pooled variance. In the case of non-normal distribution, a Kruskal–Wallis test was performed, followed by Dunn’s multiple comparison test.

## 3 Results

### 3.1 PRG5 forms homo dimers

**FIGURE 1:**
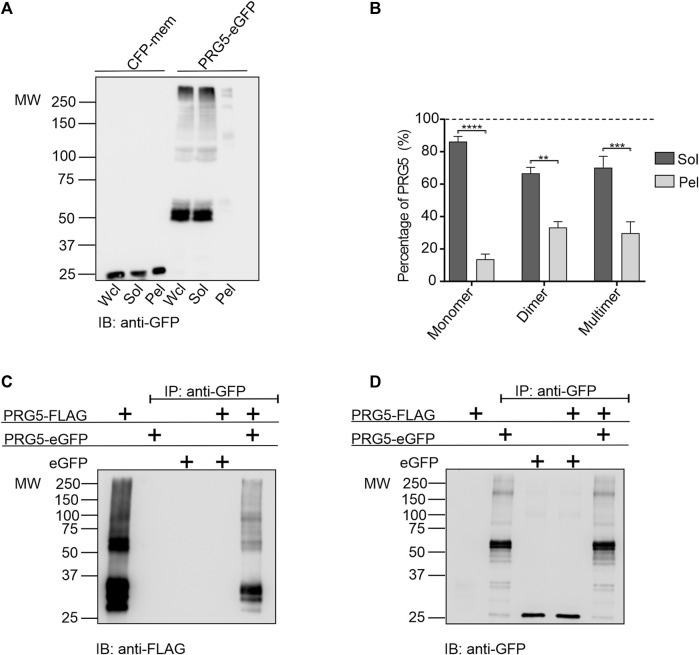
PRG5 forms homo dimers**. (A)** Solubility assay of either CFP-mem or PRG5-eGFP, showing whole cell lysate (Wcl), detergent-soluble (Sol), or insoluble pellet (Pel) fractions. **(B)** Quantification of the solubility assay of three independent repeats. The graph compares the monomeric, dimeric, and multimeric fractions of PRG5 within the Sol (dark grey) and Pel (light grey) fractions. Therefore, we set the sum of the Sol and Pel fractions to 100%. Values are given in percent. Ordinary one-way ANOVA (*p* < 0.0001) followed by Sidak’s multiple comparison test; n = 3. Graph represents mean ± SEM (Supplementary Table S1). Western blots of co-transfected PRG5-FLAG with either PRG5-eGFP or eGFP were stained with either anti-FLAG **(C)** or anti-GFP **(D)**. IP: immunoprecipitation, MW: molecular weight, IB: immunoblot.

Previous Western blot analyses have demonstrated that PRG5 is not solely present as a monomeric 35 kDa band, but also as higher molecular weight bands, which may represent potential multimeric versions. While in isolated samples obtained from PRG5 overexpressing HEK293T cells the presence of PRG5 in a higher molecular weight band was confirmed by mass spectrometry. In addition, analysis of endogenous protein lysates from cerebellum, cortex, hippocampus and bulbus revealed higher molecular weight bands representing the potential multimeric version of PRG5, which even increased with prolonged development (Gross et al., 2022a). To distinguish if these high molecular bands are multimeric versions of PRG5 or present as insoluble aggregates, we first performed an *in vitro* solubility assay. Here, we separated detergent-soluble and insoluble PRG5-eGFP fractions on Western blot. In both, the whole cell lysate fraction (Wcl) and in the detergent-soluble fraction (Sol), we detected two strong double bands around 60 kDa, which are equivalent to the predicted molecular weight of PRG5 attached to eGFP. Additionally, we found two double bands around 100 kDa, representing a potential dimeric, as well as a band above 250 kDa, representing a potential multimeric version of PRG5-eGFP. By contrast, only faint bands at 60, 100 and 250 kDa were present in the insoluble pellet fraction (Pel). In the cyan fluorescent protein (CFP)-MEM control (membrane targeted version of CFP), a 25 kDa band could be detected in all three fractions (Figure 1A). To compare how much PRG5 protein is present in the detergent-soluble and the insoluble fraction, we set the sum of the Sol and Pel fractions to 100%. We showed that 80% monomeric PRG5 is present in the soluble fraction, while 20% is insoluble. The dimeric fraction is 66% soluble and 24% insoluble, while 70% of the higher PRG5 multimers are soluble and 30% insoluble (Figure 1B). In general, the soluble PRG5 content was significantly higher than the insoluble content, suggesting that the appearance of these high molecular weight bands cannot be explained by the formation of insoluble aggregates (Supplementary Table S1). The presence of two double bands can be explained by glycosylation. Previous studies showed a consensus N-glycosylation site at amino acid 158 within the second extracellular loop of rat and mouse PRG5, which was further confirmed in endogenous cortex lysates (Coiro et al., 2014; Gross et al., 2022a). Here we demonstrated that the band shift after hydrolyzation of N-linked glycan chains by PNGase F can also be found in higher molecular weight bands between 100 and 150 kDa (Supplementary Figure S1). To analyze potential PRG5 homo multimerization, we performed co-immunoprecipitation experiments. In HEK293T cells, PRG5-eGFP and PRG5-FLAG were co-transfected and purified using GFP-specific beads. As a control, PRG5-FLAG was transfected with eGFP only. Using a FLAG-specific antibody, we show that PRG5-eGFP, but not eGFP alone, was able to co-precipitate with PRG5-FLAG, confirming the existence of at least PRG5 homo dimers (Figure 1C). As control, all three constructs were individually expressed, showing the expected band pattern. PRG5-FLAG shows three monomeric bands between 30 and 37 kDa and two double bands slightly above 50 kDa (dimer) (Figure 1C). PRG5-GFP shows two double bands at 60 kDa (monomer), and a faint band between 150 and 250 kDa (dimer), while eGFP shows a band at 25 kDa (Figure 1D).

### 3.2 PRG5 multimers are located at the plasma membrane of HEK293T cells

**FIGURE 2:**
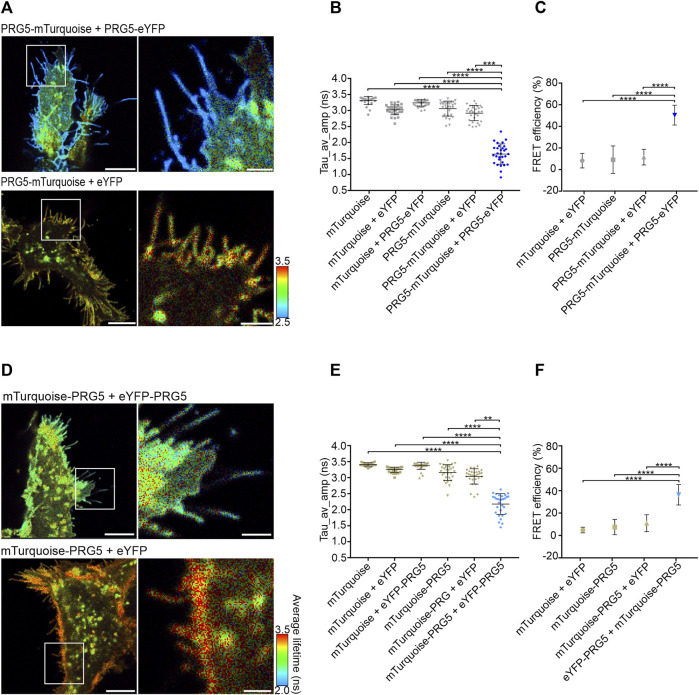
PRG5 multimers are located at the plasma membrane of HEK293T cells. Donor and acceptor are fused to either the C-terminus (**A–C**) or the N-terminus (**D, E**) of PRG5. HEK293T cells transfected with PRG5 show multimerization of PRG5 predominantly at the plasma membrane in parts with and without filopodia (lower fluorescence lifetime = blue color). This lifetime decrease was quantified by analyzing the amplitude-weighted average lifetime at the plasma membrane. The PRG5- FLIM-FRET pair (C-terminal tagged = dark blue; N-terminal tagged = light blue) showed significantly shorter fluorescence lifetimes (tau_Average_Intensity in nanoseconds) and corresponding increase in FRET efficiency (%) regardless of the localization of the fluorescent tag. Kruskal–Wallis test (*p* < 0.0001) followed by a *post hoc* Dunn’s multiple comparison test; n = 3, N = 30. Scale bars: 8 μm; zoomed images 2 µm. Tau_av_amp = Tau average amplitude. Graphs represent mean ± SEM, for *p*-values see Supplementary Tables S2–S5).

After we demonstrated the presence of PRG5 multimers, we aimed to further characterize their specific localization. PRG5 overexpression induces the formation of plasma membrane protrusions in P19 carcinoma, N1E-115 neuroblastoma, and HEK293H cells (Broggini et al., 2010; Gross et al., 2022b). Here, we used FLIM-FRET to quantify PRG5 interaction in living HEK293T cells at the plasma membrane, which comprised parts with and without filopodia. In general, FLIM-FRET measures a shorter fluorescence decay of a FRET donor only if a fluorophore acceptor comes in close proximity (<10 nm). Therefore, lower donor lifetimes and the resulting higher FRET efficiencies directly report quantitatively on PPIs (Godet and Mély, 2019; Liput et al., 2020). To investigate the effect of fluorophore localization on the efficiency of the energy transfer, donor and acceptor were fused to the N- and C-terminus of PRG5, respectively. Protein expression of all constructs was confirmed by Western blot (Supplementary Figure S2). In living HEK293T cells, co-expressed C-terminally tagged PRG5-mTurquoise and PRG5-eYFP resulted in shorter fluorescence lifetimes (visualized in blue) at the plasma membrane (Figure 2A). This was further confirmed by quantifying the amplitude-weighted average lifetime (Tau_av_amp) in nanoseconds (ns). Although there were statistically significant differences between some controls, such as between mTurquoise and mTurquoise + eYFP, the variance between the controls was less than 0.4 ns. Importantly, each individual control showed a significant decrease in the fluorescence lifetime of more than 1 ns relative to the PRG5 donor and acceptor pair. Reasons for the variability between the controls include the occurrence of bystander FRET, homo-FRET, and the process of fluorophore tagging itself (further explanations are given in the discussion). (Figure 2B; Supplementary Table S2). This lifetime decrease was further reflected in the calculation of FRET efficiency. Co-expression of PRG5-mTurquoise and PRG5-eYFP resulted in a significantly increased efficiency (50%), which was approximately five times higher compared to the controls. In addition, there were no significant differences in FRET efficiency between controls (Figure 2C; Supplementary Table S3). Similar, although smaller effects, were observed for N-terminally tagged constructs. Here, shorter lifetimes could be visualized at the plasma membrane when mTurquoise-PRG5 and eYFP-PRG5 were co-expressed (Figure 2D). Despite statistically significant differences between some controls, the variance between controls was again less than 0.4 ns. Similar to the C-terminal constructs, each individual control showed at least 1 ns longer fluorescence lifetimes when compared to the PRG5 donor- and acceptor pair (Figure 2E; Supplementary Table S4). This was likewise reflected in the FRET efficiency, where co-expression of mTurquoise-PRG5 and eYFP-PRG5 resulted in an elevated efficiency (36%), approximately threefold higher than that of the controls (Figure 2F; Supplementary Table S5). In addition, we co-expressed C-terminally tagged PRG5-mTurquoise together with the N-terminally tagged eYFP-PRG5. We found a significant increase of the amplitude-weighted average lifetime when compared with the C-terminally tagged PRG5 donor and acceptor pair. This increase was also observed when compared with the N-terminally tagged pair, although not significant. Further, we showed a significant difference of PRG5-mTurquoise + eYFP-PRG5 between the N-terminal control mTurquoise-PRG5 + eYFP, which was, however, not observed when compared to the C-terminal control PRG5-mTurquoise + eYFP (Supplementary Figure S3; Supplementary Table S11). Based on the higher fluorophore lifetime of the co-expressed C-terminal donor and N-terminal acceptor, it can be assumed that in the multimeric PRG5 complex the N- and C-terminal ends are further away from each other than the respective N-N and C-C terminal pairs. These results underline the high sensitivity of the FLIM-FRET method, which depend on parameters such as distance and fluorophore flexibility (Tregidgo et al., 2008; Liput et al., 2020). Taken together, our results provide clear evidence for the presence of PRG5 homo multimers at the plasma membrane of living HEK293T cells in parts with and without membrane filopodia.

### 3.3 PRG5 multimers are specifically localized in membrane filopodia of HEK293T cells

**FIGURE 3:**
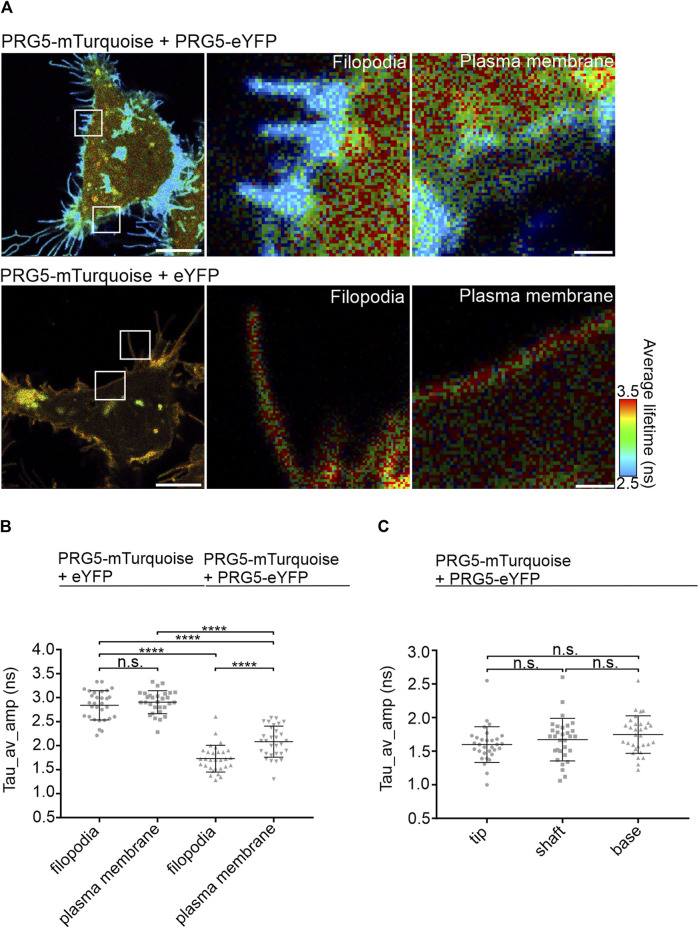
PRG5 multimers are specifically localized in membrane filopodia of HEK293T cells**. (A)** Region-specific FLIM-FRET measurements of membrane parts with or without filopodia in HEK293T, cells transfected with C-terminally tagged PRG5 FLIM-FRET pair. Shorter lifetimes are visualized in blue. **(B)** Lifetimes are quantified by analyzing the amplitude-weighted average lifetime (Tau_av_amp in nanoseconds) **(C)** Detailed analysis of filopodial tip, shaft, and base. Kruskal–Wallis test (*p* 0.0193) followed by Dunn’s multiple comparison test; n = 3, N = 30. Scale bars: 8 μm, zoomed images: 1 µm. Tau_av_amp = Tau average amplitude. Graphs represent mean ± SEM, for *p*-values see Supplementary Tables S6, S7).

To further specify the localization of PRG5 multimers, we measured the average-weighted lifetime in membrane regions with and without filopodia. In general, filopodia are defined as thin “needle-like” extensions of different length and width (Etienne-Manneville and Hall, 2002; Broggini et al., 2010). Having shown that the FRET efficiency of the C-terminally tagged constructs is higher than that of the N-terminally tagged constructs, we continued our studies with the C-terminally tagged PRG5-mTurquoise and PRG5-eYFP. In addition, we chose PRG5-mTurquoise + eYFP as control, as this showed the lowest lifetime of all controls (Figure 2B). As shown in Figure 3A, shorter lifetimes, indicated in blue, are specifically observed within the filopodia. This was further reflected in the quantification, where the lifetime in membrane parts with filopodia was significantly shorter than in regions without filopodia. However, the general lifetimes of the PRG5 donor- and acceptor pair were still significantly shorter compared to the filopodia and plasma membrane lifetimes of the control PRG5-mTurquoise + eYFP (Figure 3B; Supplementary Table S6). Next, we analyzed the lifetime distribution within the filopodia by dividing it into three different sections: tip, shaft, and base (For description of the quantification see Supplementary Figure S4). Our findings revealed a trend towards shorter lifetimes at the distal end of the filopodia, which was, not significant (Figure 3C; Supplementary Table S7).

### 3.4 PRG5 multimers are specifically localized at the tip of spine-like structures in primary hippocampal neurons

**FIGURE 4:**
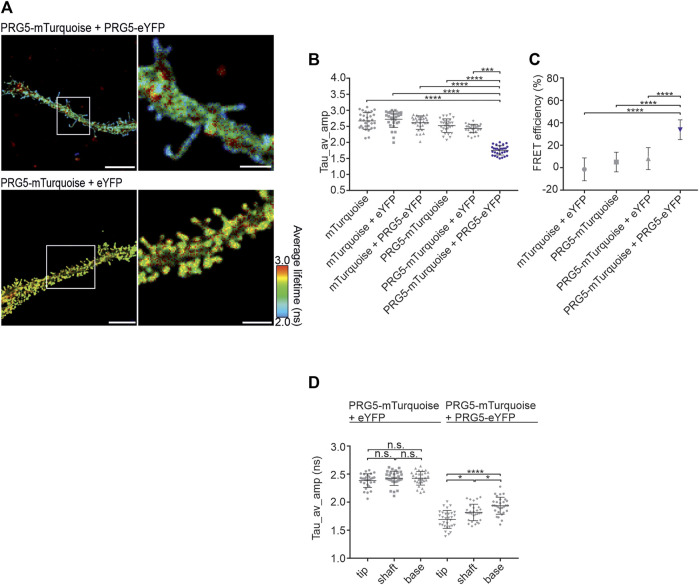
PRG5 multimers are specifically localized at the tip of spine-like structures in primary hippocampal neurons**. (A)** DIV2 primary hippocampal neurons showed a reduced lifetime (in blue) in spine-like membrane structures when PRG5-mTurquoise and PRG5-eYFP are co-expressed. **(B)** The PRG5- FLIM-FRET pair (blue) showed significantly shorter fluorescence lifetimes (tau_Average_Intensity in nanoseconds). **(C)** This was further reflected in an increase in FRET efficiency (%). **(D)** Detailed analysis of the spine-like membrane structures showed shorter lifetime towards the tip. Statistical testes: B and **(C)** Kruskal–Wallis test (*p* < 0.0001) followed by a *post hoc* Dunn’s multiple comparison test, **(D)** Ordinary one-way ANOVA (*p* < 0.0001) followed by Tukey’s multiple comparisons test. N = 3, N = 30. Scale bars: PRG5-mTurquoise + PRG5 eYFP 10 μm, PRG5-mTurquoise + eYFP 7 μM; zoomed images 3 µm. Tau_av_amp = Tau average amplitude. Graphs represent mean ± SEM, for *p*-values see Supplementary Tables S8–S10).

Given the prominent neuronal expression of endogenous PRG5 (Coiro et al., 2014; Gross et al., 2022a; Polyzou et al., 2024), we continued the analysis in fixed DIV2 primary hippocampal neurons. Overexpression of PRG5 results in a prominent enrichment at the plasma membrane in both proximal and distal regions of neurites and leads to premature formation of spine-like structures that are typically absent at DIV2. In addition, PRG5 was shown to be located specifically at the tip of each protrusion (Coiro et al., 2014). In line with these results, we showed that PRG5 overexpression leads to the formation of spine-like membrane structures and observed that shorter fluorescence lifetimes (in blue) were localized more towards the end of these protrusions (Figure 4A). Quantification of the amplitude-weighted average lifetime of the C-terminally tagged PRG5 donor and acceptor pair at the plasma membrane revealed a significantly shorter lifetime compared to the controls (Figure 4B; Supplementary Table S8). This result was further reflected in the FRET efficiency, where co-expressed PRG5-mTurquoise and PRG5-eYFP showed a significantly higher efficiency (33%) compared to the controls (Figure 4C; Supplementary Table S9). To further characterize the specific localization of PRG5 multimers in neuronal spine-like structures, we again subdivided the protrusion into tip, shaft, and base (For description of the quantification see Supplementary Figure S4). Notably, we found the shortest lifetime at the tip of spine-like structures, while it increased stepwise towards the base of the protrusion. This effect was not observed in control neurons expressing PRG5-mTurquoise + eYFP (Figure 4D; Supplementary Table S10). These results demonstrate that PRG5 multimers are predominantly localized towards the tip of spine-like structures in DIV2 primary hippocampal neurons.

## 4 Discussion

In this study, we verified the existence of functional PRG5 multimers at the plasma membrane, specifically in non-neuronal filopodia in HEK293T cells and at the tip of neuronal spine-like structures of primary hippocampal neurons. While hetero multimerization of PRG5 with its family members PRG2 and PRG3 has been shown (Yu et al., 2015), the existence of PRG5 homo multimers was not yet clarified. By using *in vitro* solubility and deglycosylation assays, we demonstrated that both monomeric and multimeric version of PRG5 were present in a soluble form and are N-glycosylated. We proved that PRG5-eGFP, but not eGFP alone, was able to co-precipitate with PRG5-FLAG, confirming the existence of at least PRG5 homo dimers. Gross and colleagues postulated PRG5 homo multimerization of endogenous PRG5 in different areas of the murine brain. When comparing Western blot results of individual brain areas, differences can be observed. For example, a higher molecular weight band, representing the potential homo multimer of PRG5, is already pronounced in cortex lysate at an early embryonic age (embryonic day 19). However, this strong expression does not occur in the cerebellum until postnatal day 15, and starts even later in the hippocampus around postnatal day 20 (Gross et al., 2022a). The results indicate the existence of disparate functions associated with monomeric and multimeric PRG5. These functions may exhibit regional variations within the brain and may even be developmentally dependent. We used FLIM-FRET to characterize the specific localization of PRG5 multimers under physiological conditions. However, care is required when analyzing FLIM-FRET experiments. First, it is important to consider the possibility of bystander FRET, which can occur, as protein overexpression can lead to molecular crowding. Bystander FRET is a non-specific form of FRET that can occur at high fluorophore concentrations. Here, acceptor tagged molecules are present at such a high concentration that wherever a donor tagged molecules is placed it will be within 10 nm (Clayton and Chattopadhyay, 2014; Liput et al., 2020). To account for this effect in our analysis, we co-expressed mTurquoise either with eYFP alone or fused to PRG5. Further it is important to exclude homo FRET between donor molecules (Liput et al., 2020), which is the reason for the inclusion of the mTurquoise control in this study. In addition, it was essential to demonstrate that fluorophore tagging itself does not impair the functionality of PRG5, or affect the flexibility of the fluorophores, so that donor and acceptor can rotate freely while attached to PRG5. This is important, given that tagging a fluorophore to a protein can itself alter its lifetime due to a change in the local refractive index (Tregidgo et al., 2008). We therefore included the controls PRG5-mTurquoise/mTurquoise-PRG5 alone, and co-expressed with eYFP, in our study. All these effects lead to a variance within the controls, which was, however, less than 0.4 ns and thus far from the actual decrease of the FLIM-FRET pairs.

Here, we demonstrated that co-expression of PRG5 donor and acceptor leads to a shorter fluorescence lifetime, regardless of localization of fluorophores. This can be particularly visualized at the plasma membrane of living HEK293T cells, in parts with and without filopodia, and confirms the existence of PRG5 homodimers. We further quantified this using the amplitude-weighted average lifetime, where we observed significantly shorter donor lifetimes at the plasma membrane when PRG5 donor and acceptor were co-expressed. By taking all our controls into account, we conclude that the donor lifetime-changes observed in our study were due to the interaction of PRG5 molecules, confirming the presence of PRG5 multimers at the plasma membrane of HEK293T cells. However, we are aware that these multimers might not of pure homogenic nature as FLIM-FRET can only detect PRG5 protein that is labeled with donor and acceptor fluorophores. Given that the existents of PRG hetero multimers is already known (Yu et al., 2015), it is likely that PRG5 further form complexes with other members of the PRG family or other yet unknown interaction partners. It is surely interesting to investigate heteromeric multimerization using FLIM-FRET. When comparing the FRET efficiencies of the N- and C-terminally tagged constructs, C-terminally tagged constructs had a higher efficiency (50%), than the N-terminally tagged constructs (36%). However, these results were expected, given that PRG5 has a long, flexible C-terminal domain, but a rather short, inflexible N-terminal domain (Brandt et al., 2024). It is likely that the short N-terminus limits the flexibility and movement of the fluorophore, which results in a more fixed distance and orientation between the donor and acceptor. In theory, this can result in either higher or lower FRET efficiency depending on whether the fixed distance falls within the Förster radius (the optimal distance for FRET) (Tregidgo et al., 2008; Caron et al., 2013; Liput et al., 2020). In case of PRG5 it led to an increased FRET-efficiency. The enhanced flexibility provided by the longer C-terminal domain is likely to enhance the interaction probability, although it may occasionally also increase the distance between the fluorescent proteins. In this case, however, it resulted in an elevated FRET efficiency. Notably, it has been shown that the fusion of eGFP to the N-terminus of PRG5 prevented its localization to the plasma membrane and, consequently, the induction of filopodia (Gross et al., 2022b). To address this issue, new constructs were designed that incorporated a spacer sequence between the fluorophores and PRG5, thereby enabling plasma membrane transport and subsequent filopodia induction. Based on these results, we continued our studies with the C-terminally tagged PRG5-mTurquoise and PRG5-eYFP. In addition, we attached our donor and acceptor to either the N- or C-terminus of PRG5, we investigated the effect of fluorophore localization on the protein. We found higher lifetimes of the N-C FLIM-FRET pair, compared to C-C and N-N, which indicates that in the multimeric PRG5 complex the N- and C-terminal ends are further away from each other than N-N and C-C terminals. To further characterize the specific localization of PRG5 multimers, we measured the amplitude-weighted average lifetime in HEK293T cells within membrane filopodia and in membrane regions without filopodia. Shorter lifetimes were specifically observable within filopodia, which was further confirmed in the quantifications. By dividing the filopodia into tip, shaft, and base (see Supplementary Figure S4 for a description of quantification), we found a trend towards shorter lifetimes at their distal end, which was however not significant.

PRG5 exhibits pronounced neuronal expression and is localized primarily in dendritic structures in *vitro* and *in vivo* experiments (Coiro et al., 2014; Gross et al., 2022a; Gross et al., 2022b). We therefore continued the study in fixed, immature primary hippocampal neurons (DIV2). In line with previous results (Broggini et al., 2010; Coiro et al., 2014), we observed a higher number of spine-like membrane structures in PRG5 over-expressing neurons, compared to controls. Here, the shorter lifetimes representing PRG5 multimers were specifically observable in these spine-like membrane structures. This was further reflected in the quantification of the amplitude-weighted average lifetime intensity of PRG5-mTurquoise and PRG5-eYFP, where significantly shorter lifetimes were detected. Notably, detailed lifetime analysis of spine-like structures revealed that PRG5 multimers were specifically localized to the distal end, rather than the shaft or base (see Supplementary Figure S4 for description of the quantification). It is of note that this effect was highly significant in neurons, while it was only a tendency in HEK239T cells. This might suggest a different functional role for PRG5 multimers in non-neuronal filopodia *versus* neuronal spine-like structures. In general, non-neuronal filopodia exhibit structural and functional differences compared to neuronal dendritic spines. Filopodia are primarily involved in cell movement and environmental sensing, while dendritic spines are specialized for synaptic connectivity and signal integration in neurons (Hering and Sheng, 2001; Segal, 2002; Mattila and Lappalainen, 2008). Therefore, it is likely, that neuronal PRG5 multimerization is influenced by neuronal molecules or factors such as signaling molecules, scaffolding proteins and/or neuro transmitter. Indeed, previous studies demonstrated the participation of PRG5 in the formation, morphology, and stabilization of dendritic spines, activity independent induction of membrane protrusions was shown. This indicates a potential role of PRG5 in spinogenesis and spine stabilization, rather than in synaptic transmission (Coiro et al., 2014; Gross et al., 2022a). However, further investigations are needed to understand the specific function of PRG5 multimers. It is possible that PRG5 multimerization is needed for the formation of membrane protrusions, or that it is necessary for the interaction of PRG5 with other molecules. For example, PRG5 is known to attenuate LPA-induced axon collapse, and it contributes to mechanisms that overcome LPA- and Neurite-growth inhibitor-A-induced neurite retraction, although it does not directly interact with LPA (Broggini et al., 2010; Coiro et al., 2014). Furthermore, RG5 interacts with different phosphorylated phosphatidylinositol phosphates at its C-terminal domain (Coiro et al., 2014). It might be possible that PRG5 multimers, are needed for this association. Although PRGs differ from LPPs due to their mutations in the catalytic domain, they are homologous. LPPs form both homo and hetero dimers, which had, however, no effect on their catalytic activity. Therefore, it is assumed that LPP multimers are responsible for regulating subcellular localization (Burnett et al., 2004; Long et al., 2008). It is thus possible that PRG5 also multimerizes to regulate its subcellular localization.

## 5 Conclusion

Our study provides compelling evidence for the presence of functional PRG5 multimers at the plasma membrane. Through *in vitro* solubility and deglycosylation assays, we established that PRG5 exists in both monomeric and multimeric forms, which are soluble and N-glycosylated. Furthermore, FLIM-FRET analysis confirmed the presence of PRG5 multimers at the plasma membrane of living HEK293T cells, specifically within filopodia. Importantly, we also identified PRG5 multimers in immature primary hippocampal neurons, where they were distinctly localized at the distal tips of spine-like structures. Interestingly, while PRG5 multimers exhibited a specific localization at the distal tips in neuronal spine-like structures, they showed only a tendency for such localization in HEK293T cell filopodia, suggesting potentially divergent functional roles in neuronal *versus* non-neuronal contexts. The ability to specifically detect PRG5 multimers opens the venue for future therapeutic strategies that may involve either disrupting or stabilizing PRG5 multimerization to improve cellular function. Future experiments may include the use of small organic fluorophores in place of traditional fluorescent proteins in FLIM-FRET. This approach could provide more detailed insights into molecular interactions by leveraging the superior photophysical properties of small organic dyes, their compatibility with super-resolution techniques, and their specific, minimalistic labeling methods. Future studies could focus on optimizing these fluorophores for FLIM-FRET and systematically comparing them with existing fluorescent protein-based approaches to determine their full potential in uncovering the multimerization dynamics of PRG5.

## Data Availability

The raw data supporting the conclusions of this article will be made available by the authors, without undue reservation.

## Abbreviations

LPP: Lipid-phosphate phosphatases, PRG: Plasticity-related genes, FLIM: Fluorescence Lifetime Imaging, FRET: Förster Resonance Energy Transfer, LPA: Lysophosphatidic Acid, PPI: Protein-protein interaction.
