# Supplementary material for: Visualizing multimerization of plasticity-related gene 5 at the plasma membrane using FLIM-FRET
Source: Front Mol Biosci. 2024 Sep 30;11:1478291. doi: 10.3389/fmolb.2024.1478291 (PMC11471602; doi:10.3389/fmolb.2024.1478291)
Supplement: Supplementary file 1 [file DataSheet1.DOCX]

Supplementary Material

## Supplementary Figures


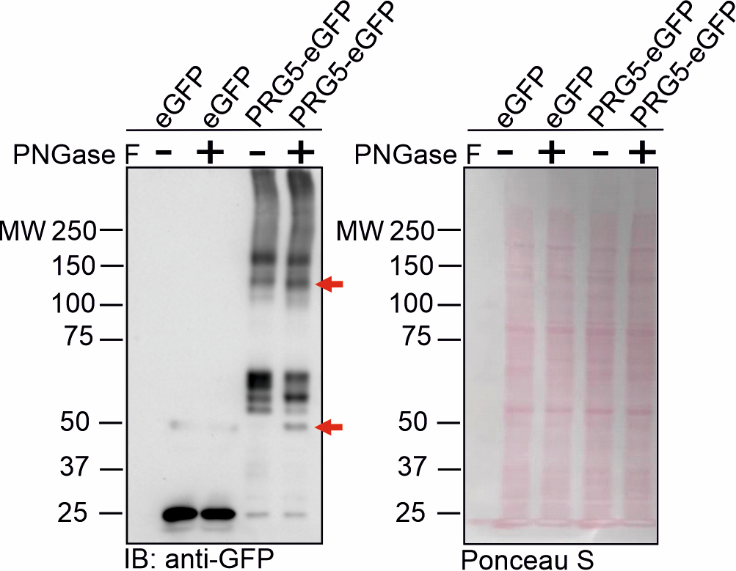


Supplementary Figure 1: PRG5 deglycosylation assay. Representative Western blot showing the band shift after hydrolysis of N-linked glycan chains using PNGase F in the monomeric 60 kDa band and in the higher molecular weight bands between 100 and 150 kDa (red arrows). Protein was visualized with anti-GFP antibody. Ponceau S staining of total protein is shown as loading control.


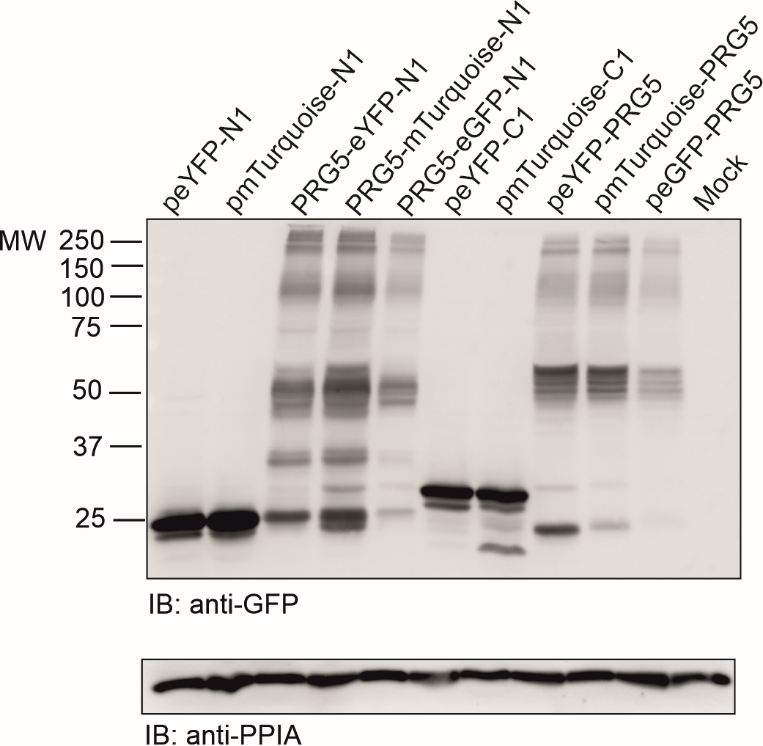


Supplementary Figure 2: Expression control of FLIM-FRET constructs. Representative Western blot showing the N- and C-terminal control eYFP and mTurquoise around 25 kDa while the N- and C-terminal PRG5 constructs show bands at 60 kDa and between 100 and 150 kDa. Anti-GFP antibody was used to detect mTurquoise and eYFP tagged constructs. Anti-PPIA was used as the house-keeping gene, running at 14 kDa.


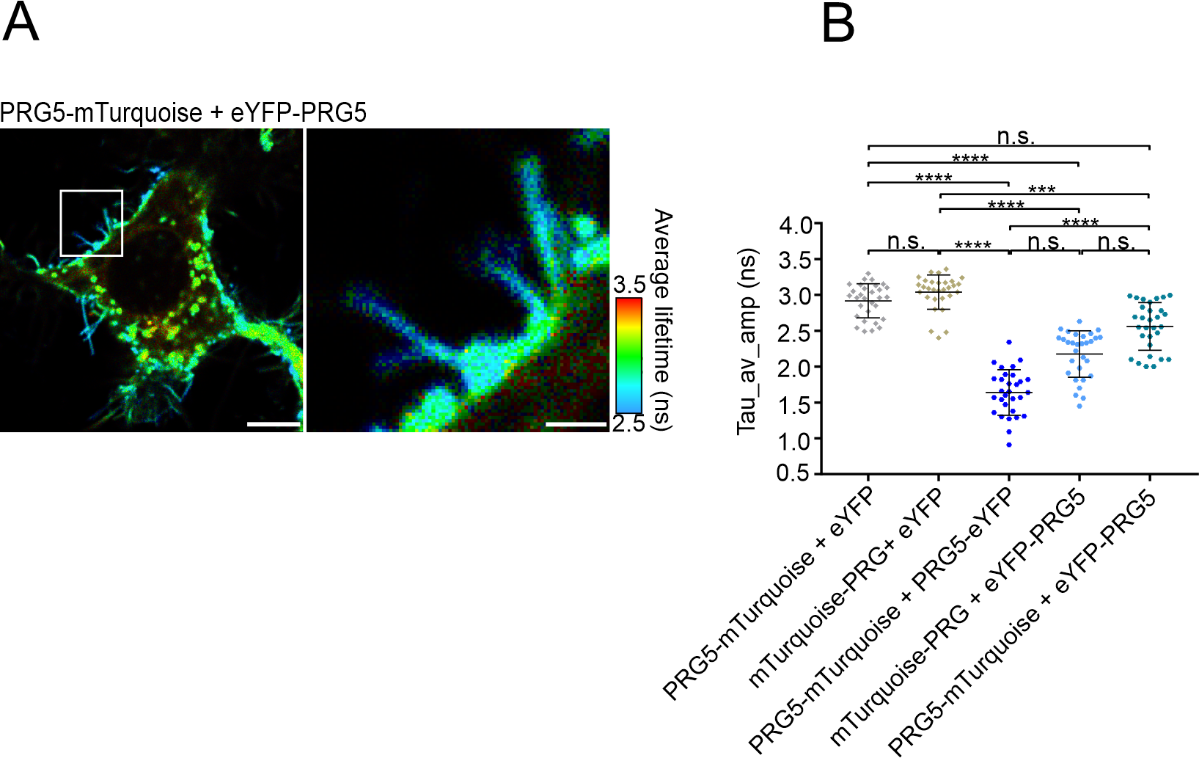


Supplementary Figure 3: FLIM-FRET measurement of C-terminally PRG5-Turquoise co-expressed with N-terminally eYFP-PRG5. (A) Representative FLIM images showing a shorter lifetime at the plasma membrane of HEK293T cells (blue). (B) Quantification of the amplitude-weighted average lifetime (Tau_av_amp) in nanoseconds (ns). For comparison, PRG5-mTurquoise + eYFP-PRG5 (petrol) were visualized using the data from Figure2B and E. C-terminally tagged constructs are shown in blue and grey, while N-terminally tagged constructs are shown in light blue and khaki. Kruskal-Wallis test (p <0.0001) followed by a post hoc Dunn’s multiple comparison test; n=3, N=30. Scale bars: 8 µm; zoomed images 2 µm. Tau_av_amp = Tau average amplitude. Graphs represent mean ± SEM, for *p*-values see Supplementary Table 11).

*Detailed protocol for protrusion quantification*

Quantifying FLIM-FRET within membrane protrusions, for example within non-neuronal filopodia or neuronal spine-like structures, requires careful considerations of several factors to maintain accuracy and reproducibility. To ensure a high-resolution image, necessary to clearly distinguish between protrusion tip, shaft, and base, we kept imaging parameters such as laser power and, detector settings, constant in all samples. As shorter lifetimes are visualized in blue, we used black and white images for all our quantifications, thus preventing potential bias in the quantification process. For the definition of the region of interests (ROIs), we carried out a morphological identification, based on size and shape. ROIs were selected manually using the paint function of the SymPho Time software. The protrusion tip was defined as the distal end of the filopodia or spine-like structure, which appeared consistent in size relative to the overall length of the protrusion. Protrusion tips were selected using circular or elliptical ROIs (**Supplementary Figure 4**, white circles). The protrusion shaft was defined by its position and uniformity. The shaft of the filopodia and spine-like structures was defined as the straight midsection between tip and base that could differ in length. Straight- to elliptical ROIs that did not extended into the ROIs of tip or base were used for selection (**Supplementary Figure 4**, yellow circles). The base of filopodia or spine-like structures were defined as part of the protrusions that connected the protrusions to the main dendritic shaft in the case of the spine-like structures, or the main part of the HEK293T cell in the case of the filopodia. These parts were often broader and more integrated when compared to the main dendrite, especially in neurons. We selected circular- to elliptical ROIs with a similar size to the tip (**Supplementary Figure 4**, green circles). To reduce variability and to ensure proper fit of the calculated lifetimes to the actual data, we always selected around 10^3^ counts per ROI.


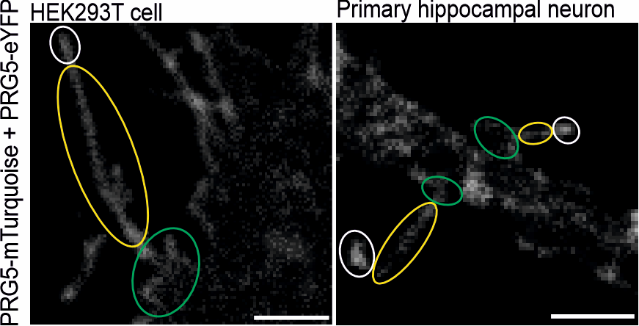


Supplementary Figure 4: Detailed protrusion quantification. Representative images used for the detailed quantification of membrane protrusions either in HEK293T cells (filopodia, left side) or in immature primary hippocampal neurons (spine-like structures, right side). Regions of interest (ROIs) are schematically indicated by circles. Distal protrusion tips are circled in white, protrusion shafts are circled in yellow, while protrusion bases are circled in green. Scale bar:2 µm.

## Supplementary Tables

Supplementary Table 1 Represents the mean difference between monomer, dimer and multimer using a Sidak’s multiple comparison test. Data represented in Figure 1B.

| Sidak's multiple comparisons test- Soluble - Pellet | Mean Difference | 95.00% Column of Difference | Significant? | Summary |
| --- | --- | --- | --- | --- |
| Monomer | 72.62 | 53.35 to 91.9 | Yes | **** |
| Dimer | 33.48 | 14.2 to 52.76 | Yes | ** |
| Multimer | 40.42 | 21.15 to 59.7 | Yes | *** |

Supplementary Table 2: Summary of *p* values of pairwise comparison by the Kruskal-Wallis test, followed by Dunn’s multiple comparison test. Data represented in Figure 2B. n.s.= non-significant

| Dunn's multiple comparisons test | Significant? | Summary | *p*-values |
| --- | --- | --- | --- |
| mTurquoise vs. mTurquoise + eYFP | Yes | **** | <0.0001 |
| mTurquoise vs. mTurquoise + PRG5-eYFP | No | n.s. | >0.9999 |
| mTurquoise vs. PRG5-mTurquoise | Yes | *** | 0.0004 |
| mTurquoise vs. PRG5-mTurquoise + eYFP | Yes | **** | <0.0001 |
| mTurquoise vs. PRG5-mTurquoise + PRG5-eYFP | Yes | **** | <0.0001 |
| mTurquoise + eYFP vs. mTurquoise + PRG5-eYFP | Yes | ** | 0.0047 |
| mTurquoise + eYFP vs. PRG5-mTurquoise | No | n.s. | >0.9999 |
| mTurquoise + eYFP vs. PRG5-mTurquoise + eYFP | No | n.s. | >0.9999 |
| mTurquoise + eYFP vs. PRG5-mTurquoise + PRG5-eYFP | Yes | **** | <0.0001 |
| mTurquoise + PRG5-eYFP vs. PRG5-mTurquoise | No | n.s. | 0.1995 |
| mTurquoise + PRG5-eYFP vs. PRG5-mTurquoise + eYFP | Yes | *** | 0.0002 |
| mTurquoise + PRG5-eYFP vs. PRG5-mTurquoise + PRG5-eYFP | Yes | **** | <0.0001 |
| PRG5-mTurquoise vs. PRG5-mTurquoise + eYFP | No | n.s. | 0.8602 |
| PRG5-mTurquoise vs. PRG5-mTurquoise + PRG5-eYFP | Yes | **** | <0.0001 |
| PRG5-mTurquoise + eYFP vs. PRG5-mTurquoise + PRG5-eYFP | Yes | *** | 0.0007 |

Supplementary Table 3: Summary of *p* values of pairwise comparison by the Kruskal-Wallis test, followed by Dunn’s multiple comparison test. Data represented in Figure 2C. n.s.= non-significant

| Dunn's multiple comparisons test | Significant? | Summary | *p*-values |
| --- | --- | --- | --- |
| mTurquoise + eYFP vs. PRG5-mTurquoise | No | n.s. | >0,9999 |
| mTurquoise + eYFP vs. PRG5-mTurquoise + eYFP | No | n.s. | >0,9999 |
| mTurquoise + eYFP vs. PRG5-mTurquoise + PRG5-eYFP | Yes | **** | <0,0001 |
| PRG5-mTurquoise vs. PRG5-mTurquoise + eYFP | No | n.s. | 0,9279 |
| PRG5-mTurquoise vs. PRG5-mTurquoise + PRG5-eYFP | Yes | **** | <0,0001 |
| PRG5-mTurquoise + eYFP vs. PRG5-mTurquoise + PRG5-eYFP | Yes | **** | <0,0001 |

Supplementary Table 4: Summary of *p* values of pairwise comparison by the Kruskal-Wallis test, followed by Dunn’s multiple comparison test. Data represented in Figure 2E. n.s.= non-significant

| Dunn's multiple comparisons test | Significant? | Summary | *p*-values |
| --- | --- | --- | --- |
| mTurquoise vs. mTurquoise + eYFP | Yes | *** | 0.0003 |
| mTurquoise vs. mTurquoise + eYFP-PRG5 | No | n.s. | >0.9999 |
| mTurquoise vs. mTurquoise-PRG5 | Yes | **** | <0.0001 |
| mTurquoise vs. mTurquoise -PRG+ eYFP | Yes | **** | <0.0001 |
| mTurquoise vs. mTurquoise-PRG5 + eYFP-PRG5 | Yes | **** | <0.0001 |
| mTurquoise + eYFP vs. mTurquoise + eYFP-PRG5 | Yes | * | 0.0113 |
| mTurquoise + eYFP vs. mTurquoise-PRG5 | No | n.s. | >0.9999 |
| mTurquoise + eYFP vs. mTurquoise -PRG+ eYFP | No | n.s. | 0.4465 |
| mTurquoise + eYFP vs. mTurquoise-PRG5 + eYFP-PRG5 | Yes | **** | <0.0001 |
| mTurquoise + eYFP-PRG5 vs. mTurquoise-PRG5 | Yes | ** | 0.0033 |
| mTurquoise + eYFP-PRG5 vs. mTurquoise -PRG+ eYFP | Yes | **** | <0.0001 |
| mTurquoise + eYFP-PRG5 vs. mTurquoise-PRG5 + eYFP-PRG5 | Yes | **** | <0.0001 |
| mTurquoise-PRG5 vs. mTurquoise -PRG+ eYFP | No | n.s. | 0.9651 |
| mTurquoise-PRG5 vs. mTurquoise-PRG5 + eYFP-PRG5 | Yes | **** | <0.0001 |
| mTurquoise -PRG+ eYFP vs. mTurquoise-PRG5 + eYFP-PRG5 | Yes | ** | 0.0095 |

Supplementary Table 5: Summary of *p* values of pairwise comparison by the Kruskal-Wallis test, followed by Dunn’s multiple comparison test. Data represented in Figure 2F. n.s.= non-significant

| Dunn's multiple comparisons test | Significant? | Summary | *p*-values |
| --- | --- | --- | --- |
| mTurquoise+eYFP vs. mTurquoise-PRG5 | No | n.s. | >0.9999 |
| mTurquoise+eYFP vs. mTurquoise-PRG5 + eYFP | Yes | * | 0.0214 |
| mTurquoise+eYFP vs. eYFP-PRG5 + mTurquoise-PRG5 | Yes | **** | <0.0001 |
| mTurquoise-PRG5 vs. mTurquoise-PRG5 + eYFP | No | n.s. | 0.4400 |
| mTurquoise-PRG5 vs. eYFP-PRG5 + mTurquoise-PRG5 | Yes | **** | <0.0001 |
| mTurquoise-PRG5 + eYFP vs. eYFP-PRG5 + mTurquoise-PRG5 | Yes | **** | <0.0001 |

Supplementary Table 6: Summary of *p* values of pairwise comparison using the ordinary one-way ANOVA, followed by Tukey’s multiple comparison test. Data represented in Figure 3B. n.s.= non-significant

| Tukey's multiple comparisons test | Significant? | Summary | *p-*values |
| --- | --- | --- | --- |
| Filopodia vs. Plasma membrane | Yes | **** | <0.0001 |
| Filopodia vs. Filopodia | Yes | **** | <0.0001 |
| Filopodia vs. Plasma membrane | Yes | **** | <0.0001 |
| Plasma membrane vs. Filopodia | Yes | **** | <0.0001 |
| Plasma membrane vs. Plasma membrane | Yes | **** | <0.0001 |
| Filopodia vs. Plasma membrane | No | n.s. | 0.8177 |

Supplementary Table 7: Summary of *p* values of pairwise comparison by the Kruskal-Wallis test, followed by Dunn’s multiple comparison test. Data represented in Figure 3C. n.s.= non-significant

| Dunn's multiple comparisons test | Significant? | Summary | *p-*values |
| --- | --- | --- | --- |
| Tip vs. Shaft | No | n.s. | 0.6292 |
| Tip vs. Base | No | n.s. | 0.0591 |
| Shaft vs. Base | No | n.s. | 0.8434 |

Supplementary Table 8: Summary of *p* values of pairwise comparison by the Kruskal-Wallis test, followed by Dunn’s multiple comparison test. Data represented in Figure 4B. n.s.= non-significant

| Dunn's multiple comparisons test | Significant? | Summary | *p-*values |
| --- | --- | --- | --- |
| mTurquoise vs. mTurquoise + eYFP | No | n.s. | >0.9999 |
| mTurquoise vs. mTurquoise + PRG5-eYFP | No | n.s. | >0.9999 |
| mTurquoise vs. PRG5-mTurquoise | No | n.s. | 0.9731 |
| mTurquoise vs. PRG5-mTurquoise + eYFP | Yes | * | 0.0139 |
| mTurquoise vs. PRG5-mTurquoise + PRG5-eYFP | Yes | **** | <0.0001 |
| mTurquoise + eYFP vs. mTurquoise + PRG5-eYFP | No | n.s. | >0.9999 |
| mTurquoise + eYFP vs. PRG5-mTurquoise | No | n.s. | 0.0531 |
| mTurquoise + eYFP vs. PRG5-mTurquoise + eYFP | Yes | *** | 0.0002 |
| mTurquoise + eYFP vs. PRG5-mTurquoise + PRG5-eYFP | Yes | **** | <0.0001 |
| mTurquoise + PRG5-eYFP vs. PRG5-mTurquoise | No | n.s. | >0.9999 |
| mTurquoise + PRG5-eYFP vs. PRG5-mTurquoise + eYFP | No | n.s. | 0.0561 |
| mTurquoise + PRG5-eYFP vs. PRG5-mTurquoise + PRG5-eYFP | Yes | **** | <0.0001 |
| PRG5-mTurquoise vs. PRG5-mTurquoise + eYFP | No | n.s. | >0.9999 |
| PRG5-mTurquoise vs. PRG5-mTurquoise + PRG5-eYFP | Yes | **** | <0.0001 |
| PRG5-mTurquoise + eYFP vs. PRG5-mTurquoise + PRG5-eYFP | Yes | *** | 0.0003 |

Supplementary Table 9: Summary of *p* values of pairwise comparison by the Kruskal-Wallis test, followed by Dunn’s multiple comparison test. Data represented in Figure 4C. n.s.= non-significant

| Dunn's multiple comparisons test | Significant? | Summary | *p-*values |
| --- | --- | --- | --- |
| mTurquoise + eYFP vs. PRG5-mTurquoise | No | n.s. | 0.2496 |
| mTurquoise + eYFP vs. PRG5-mTurquoise + eYFP | Yes | * | 0.0225 |
| mTurquoise + eYFP vs. PRG5-mTurquoise + PRG5-eYFP | Yes | **** | <0.0001 |
| PRG5-mTurquoise vs. PRG5-mTurquoise + eYFP | No | n.s. | >0.9999 |
| PRG5-mTurquoise vs. PRG5-mTurquoise + PRG5-eYFP | Yes | **** | <0.0001 |
| PRG5-mTurquoise + eYFP vs. PRG5-mTurquoise + PRG5-eYFP | Yes | **** | <0.0001 |

Supplementary Table 10: Summary of *p* values of pairwise comparison by the Kruskal-Wallis test, followed by Dunn’s multiple comparison test. Data represented in Figure 4D. n.s.= non-significant

| Dunn's multiple comparisons test | Significant? | Summary | *p*-values |
| --- | --- | --- | --- |
| PRG5-mTurquoise + eYFP Tip vs. PRG5-mTurquoise + eYFP Tip Shaft | No | n.s**.** | >0.9999 |
| PRG5-mTurquoise + eYFP Tip vs. PRG5-mTurquoise + eYFP Tip Base | No | n.s. | >0.9999 |
| PRG5-mTurquoise + eYFP Tip vs. PRG5-mTurquoise + PRG5-eYFP Tip | Yes | **** | <0.0001 |
| PRG5-mTurquoise + eYFP Tip vs. PRG5-mTurquoise + PRG5-eYFP Shaft | Yes | **** | <0.0001 |
| PRG5-mTurquoise + eYFP Tip vs. PRG5-mTurquoise + PRG5-eYFP Base | Yes | **** | <0.0001 |
| PRG5-mTurquoise + eYFP Tip Shaft vs. PRG5-mTurquoise + eYFP Tip Base | No | n.s. | >0.9999 |
| PRG5-mTurquoise + eYFP Tip Shaft vs. PRG5-mTurquoise + PRG5-eYFP Tip | Yes | **** | <0.0001 |
| PRG5-mTurquoise + eYFP Tip Shaft vs. PRG5-mTurquoise + PRG5-eYFP Shaft | Yes | **** | <0.0001 |
| PRG5-mTurquoise + eYFP Tip Shaft vs. PRG5-mTurquoise + PRG5-eYFP Base | Yes | **** | <0.0001 |
| PRG5-mTurquoise + eYFP Tip Base vs. PRG5-mTurquoise + PRG5-eYFP Tip | Yes | **** | <0.0001 |
| PRG5-mTurquoise + eYFP Tip Base vs. PRG5-mTurquoise + PRG5-eYFP Shaft | Yes | **** | <0.0001 |
| PRG5-mTurquoise + eYFP Tip Base vs. PRG5-mTurquoise + PRG5-eYFP Base | Yes | **** | <0.0001 |
| PRG5-mTurquoise + PRG5-eYFP Tip vs. PRG5-mTurquoise + PRG5-eYFP Shaft | No | n.s. | >0.9999 |
| PRG5-mTurquoise + PRG5-eYFP Tip vs. PRG5-mTurquoise + PRG5-eYFP Base | No | n.s. | 0.1154 |
| PRG5-mTurquoise + PRG5-eYFP Shaft vs. PRG5-mTurquoise + PRG5-eYFP Base | No | n.s. | >0.9999 |

Supplementary Table 11: Summary of *p* values of pairwise comparison by the Kruskal-Wallis test, followed by Dunn’s multiple comparison test. Data represented in Supplementary Figure 3. n.s.= non-significant

| Dunn's multiple comparisons test | Significant? | Summary | *p*-values |
| --- | --- | --- | --- |
| PRG5-mTurquoise + PRG5-eYFP vs. mTurquoise-PRG + eYFP-PRG5 | No | n.s. | 0.1159 |
| PRG5-mTurquoise + PRG5-eYFP vs. PRG5-mTurquoise + eYFP-PRG5 | Yes | **** | <0.0001 |
| PRG5-mTurquoise + PRG5-eYFP vs. PRG5-mTurquoise + eYFP | Yes | **** | <0.0001 |
| PRG5-mTurquoise + PRG5-eYFP vs. mTurquoise-PRG+ eYFP | Yes | **** | <0.0001 |
| mTurquoise-PRG + eYFP-PRG5 vs. PRG5-mTurquoise + eYFP-PRG5 | No | n.s. | 0.0807 |
| mTurquoise-PRG + eYFP-PRG5 vs. PRG5-mTurquoise + eYFP | Yes | **** | <0.0001 |
| mTurquoise-PRG + eYFP-PRG5 vs. mTurquoise-PRG+ eYFP | Yes | **** | <0.0001 |
| PRG5-mTurquoise + eYFP-PRG5 vs. PRG5-mTurquoise + eYFP | No | n.s. | 0.0503 |
| PRG5-mTurquoise + eYFP-PRG5 vs. mTurquoise-PRG+ eYFP | Yes | *** | 0.0009 |
| PRG5-mTurquoise + eYFP vs. mTurquoise-PRG+ eYFP | No | n.s. | >0.9999 |
